# Single-session endoscopic ultrasound-guided tissue acquisition followed by choledochoduodenostomy in a patient with Roux-en-Y reconstruction

**DOI:** 10.1055/a-2368-4205

**Published:** 2024-08-12

**Authors:** Tesshin Ban, Yoshimasa Kubota, Naoto Imura, Shun Sasoh, Takashi Joh

**Affiliations:** 1 36884 Department of Gastroenterology and Hepatology, Gamagori City Hospital, Gamagori, Japan

Endoscopic ultrasound-guided tissue acquisition (EUS-TA) through the afferent limb of a Roux-en-Y reconstruction is possible but challenging 1 2 . In reconstructed patients with subsequent distal malignant biliary obstruction, EUS-TA with endoscopic ultrasound-guided choledochoduodenostomy (EUS-CDS) potentially achieves both pathological diagnosis and biliary drainage in a single session.

A 78-year-old man, with a history of total gastrectomy 8 years previously for poorly differentiated gastric adenocarcinoma, was admitted with distal malignant biliary obstruction and acute cholangitis possibly due to post-gastrectomy lymph node recurrence or stage III pancreatic cancer ( Fig. 1 ). Single-session EUS-TA followed by EUS-CDS was performed for pathological diagnosis and biliary drainage ( Video 1 ). First, we inserted a balloon-assisted endoscope (EI-580BT; Fujifilm, Tokyo, Japan) into the end of the afferent limb. Second, we placed a 0.035-inch ultra-stiff guidewire (Wrangler SUS, Piolax, Yokohama, Japan) through the surgically altered intestine for echoendoscope navigation. Third, an oblique-viewing echoendoscope (EG-580UT, Fujifilm) was advanced to around the pancreatic head using the over-the-guidewire technique 2 . Fourth, EUS-TA was performed on the mass using a 22-gauge Franseen needle ( Fig. 2 **a** ). Finally, we attempted EUS-CDS as follows: biliary puncture using a 19-gauge lancet needle, 0.025-inch guidewire placement, electrocautery anastomosis dilation, and deployment of a covered self-expandable metallic stent, 10mm in diameter and 8cm in length ( Fig. 2 **b** ). The whole clinical course was uneventful. The patient recovered from acute cholangitis and received appropriate chemotherapy following a definitive diagnosis of well-differentiated pancreatic adenocarcinoma, ruling out lymph node recurrence from the previous gastric cancer ( Fig. 3 ).

**Fig. 1:**
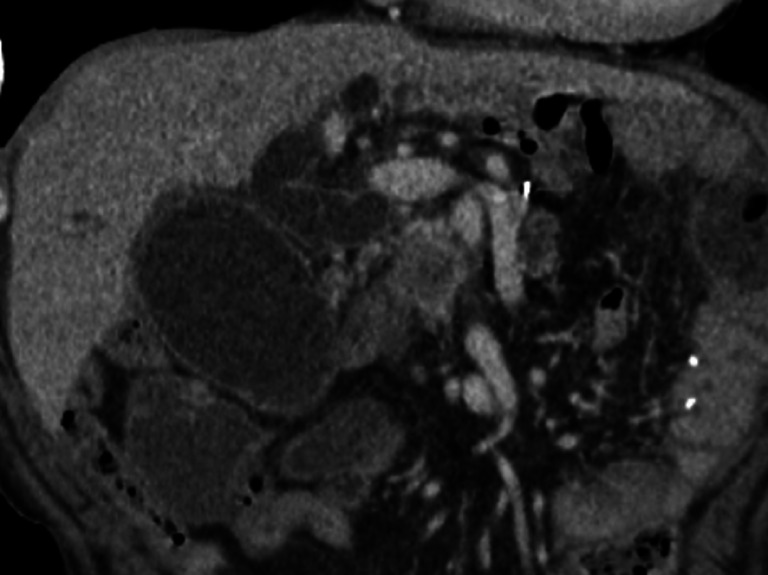
Contrast-enhanced computed tomography (CT) image in coronal section. CT reveals a hypodense mass with a hyperdense rim around the pancreatic head.

Single-session endoscopic ultrasound-guided tissue acquisition followed by choledochoduodenostomy in a patient with Roux-en-Y reconstruction.Video 1

**Fig. 2:**
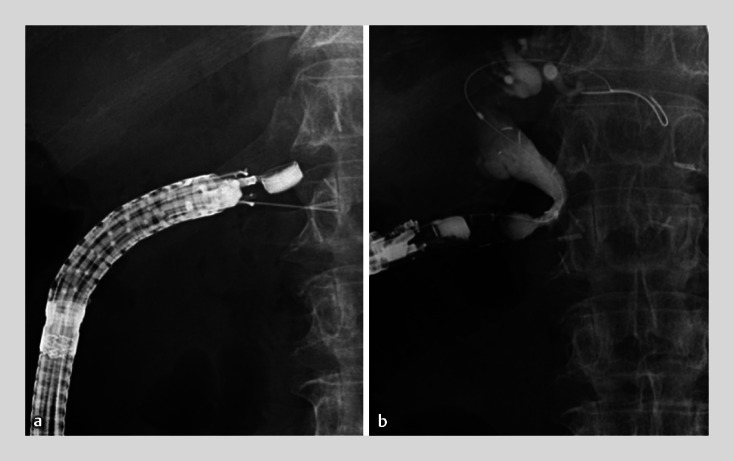
Endoscopic ultrasound-guided tissue acquisition followed by choledochoduodenostomy via the afferent limb. **a** Endoscopic ultrasound-guided tissue acquisition is performed using a 22-gauge Franseen needle. **b** Following endoscopic ultrasound-guided choledochoduodenostomy, a covered self-expandable metallic stent is deployed.

**Fig. 3:**
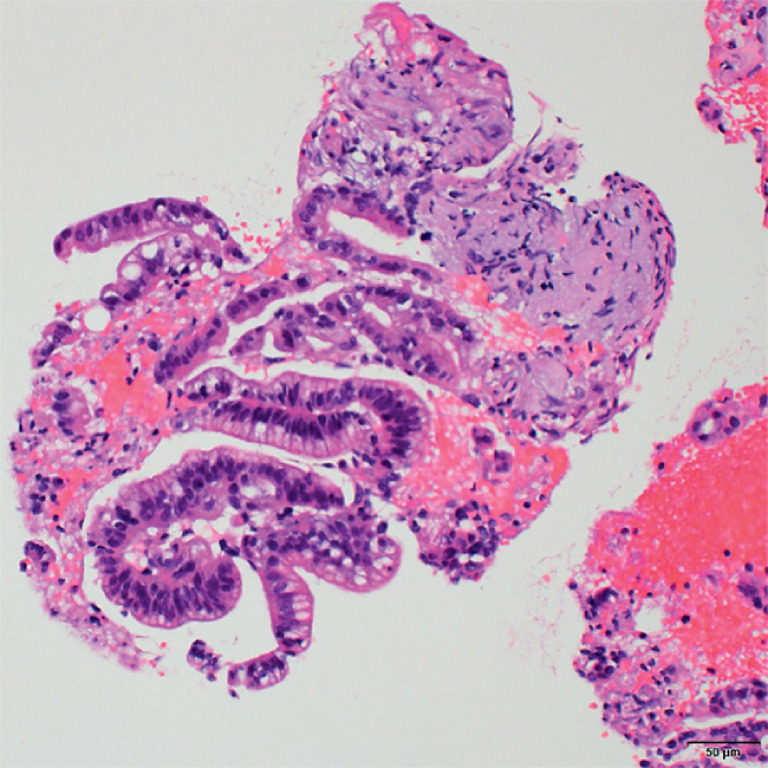
Specimen obtained by endoscopic ultrasound-guided tissue acquisition. The specimen shows well-differentiated adenocarcinoma with pancreatic stroma and no involvement of lymph node structures, distinguishing it from lymph node metastasis of poorly differentiated gastric adenocarcinoma.

In patients with Roux-en-Y reconstruction and distal malignant biliary obstruction, tissue acquisition, and biliary drainage via the endoscopic ultrasound-guided hepaticogastric route is an alternative 3 4 . However, intraductal biliary tissue acquisition has an inferior diagnostic yield compared with that of EUS-TA 5 . In this case, EUS-TA combined with EUS-CDS through the afferent limb facilitated simultaneous pathological diagnosis and drainage.

Endoscopy_UCTN_Code_TTT_1AS_2AF

## Notes

Endoscopy E-Videos https://eref.thieme.de/e-videos
*E-Videos* is an open access online section of the journal *Endoscopy* , reporting on interesting cases and new techniques in gastroenterological endoscopy. All papers include a high-quality video and are published with a Creative Commons CC-BY license. Endoscopy E-Videos qualify for HINARI discounts and waivers and eligibility is automatically checked during the submission process. We grant 100% waivers to articles whose corresponding authors are based in Group A countries and 50% waivers to those who are based in Group B countries as classified by Research4Life (see: https://www.research4life.org/access/eligibility/ ). This section has its own submission website at https://mc.manuscriptcentral.com/e-videos .
